# Physical Activity in Cancer Rehabilitation and Technology Acceptance: Results From the “Oncology in Motion” Project

**DOI:** 10.37825/2239-9747.1063

**Published:** 2024-10-01

**Authors:** Helena Biancuzzi, Francesca Dal Mas, Maurizio Massaro, Beatrice Apicerni, Lorenzo Cobianchi, Rym Bednarova, Giulia Bongiorno, Alessandro Vittori, Marco Cascella, Luca Miceli

**Affiliations:** a Department of Economics, Università Ca’ Foscari, Venice, Italy; b Department of Management, Università Ca’ Foscari, Venice, Italy; c Collegium Medicum, University of Social Sciences, Łodz, Poland; d Centro di Riferimento Oncologico di Aviano (CRO) IRCCS, Aviano (PN), Italy; e Department of Clinical, Surgical, Diagnostic & Pediatric Sciences, University of Pavia, Pavia, Italy; f General Surgery Department, Fondazione IRCCS Policlinico San Matteo, Pavia, Italy; g Department of Pain Medicine, Hospital of Latisana, Latisana, Italy; h “Friuli Riabilitazione” Rehabilitation Center, Roveredo in Piano, Italy; i Department of Anesthesia and Critical Care, ARCO ROMA, Ospedale Pediatrico Bambino Gesù IRCCS Rome, Italy; j Department of Medicine, Surgery and Dentistry, University of Salerno, 84081, Baronissi, Salerno, Italy

**Keywords:** Cancer, Pain, Telemedicine, Rehabilitation, Breast, Surgery

## Abstract

**Aims:**

The international literature underlines that physical activity has a role in preventing cancer and is beneficial for cancer recovery and rehabilitation. Therefore, patient education is essential to stimulate training. Telemedicine and e-health tools like apps and wearables can support patients’ education and the monitoring of their health condition and progress.

**Methods:**

The article reports the results of the Oncology in Motion telemedicine program of the National Cancer Institute of Aviano, Italy, to provide breast cancer patients with a personalized fitness path and telemonitoring.

**Results:**

144 women took part in the program. Low adherence was recorded, performing the customized training schedule and, for those women sticking to the plan, using the technological devices and submitting the training data to the Institute.

**Conclusion:**

Low technology acceptance and literacy, laziness, and lack of collaboration between cancer centers stood among the causes of low adherence, calling for more comprehensive and effective educational programs and support to stimulate physical activity and the use of new devices to get personalized counseling and contribute to the creation of knowledge.

## 1. Introduction

Cancer stands as a leading cause of death worldwide, accounting for nearly 10 million deaths in 2020. The most common neoplasm in 2020, in terms of new cancer cases, was breast cancer, with 2.26 million cases [1]. In 2018, the American College of Sports Medicine (ACSM) updated its guidelines to exercise for the prevention of cancer, as well as for the prevention and treatment of its effects (e.g., fatigue, anxiety, depression, general impact on the quality of life) [2]. The results of the ACSM roundtable support the existence of several biologically plausible mechanisms by which physical activity may influence cancer risk and that it is beneficial for the prevention of several types of cancer, including breast cancer, colon, endometrium, kidney, bladder, esophagus, and stomach. It is therefore strongly recommended that sedentary lifestyles be reduced to a minimum [1,3].

Despite these guidelines and the numerous studies in the literature, most people affected by cancer or who survive it are not regularly physically active [2,4]. This is why the oncological path, which also includes the post-surgical phase, home rehabilitation, and resumption of daily routines, presents the continuous need for studies, improvements, and patients’ educational programs, encouraging and implementing dedicated projects toward constant physical activity.

Furthermore, following the COVID-19 pandemic, telemedicine and e-health tools have become more widespread, also following an increase in the digital literacy of the general population. The recent pandemic has highlighted the need for innovative care pathways to reduce face-to-face appointments [5], stimulating teleconsultations, tele-visits, and telemonitoring activities [6], also following a virtual hospital model of care [7]. Several recent studies report the use of wearable devices [5,8] as an innovative method of monitoring patients, for example, after oncological surgery [9–13]. Such devices allow the monitoring of physical activity parameters, like the number of daily steps and hours of sleep, and physiological data, such as heart and respiratory rates. Wearable devices like smartwatches are, therefore, particularly valuable to constantly monitor patients and trace their personalized recovery path [14,15]. They also have the potential to predict or detect the onset of postoperative complications and can also be an opportunity to involve patients as active participants in their healing phase, transforming the process into a co-production journey [16,17].

Indeed, in co-production, patients, as healthcare service users, contribute to creating value for themselves by actively participating in their healing and rehabilitation journey. While co-production stands today as one of the most interesting and effective strategies of the modern healthcare scenario [18,19], it needs proper tools and facilitators to be fully implemented and to translate the required knowledge from the medical team to empowered patients [20,21].

Wearable devices and their use as instruments to support patients’ healing journeys have been at the center of several investigations. It should be noted that existing studies involve an extensive range of wearable devices and have employed heterogeneous data collection and analysis methods. This makes it difficult to generalize the strengths and weaknesses of the tools and, more importantly, the acceptance rates of patients in the use of the new technology [22]. Indeed, to our knowledge, very few studies evaluate the acceptance of wearable devices by breast-operated patients. Among these, Kokts-Porietis et al. [23]underline how smartwatches can be perceived as effective facilitators of physical activity. Still, technological barriers arise in their use and adherence to the defined fitness plan. Nguyen, Nga et al. [24] studied the impacts of using different types of smartwatches on women with breast cancer. Encouraging results emerged, as these devices proved to be very effective even on a population that may not be too young and not always familiar with the latest technologies. However, the investigation underlined the importance of technical support for installing and using the devices and their services, including medical consultation.

The opportunities offered by the most innovative tools in cancer recovery and education and the lack of common paradigms call for further studies and investigations that can deepen the eventual issues in the use of wearable devices concerning the oncological pathology treated and the characteristics of the patients (including age, cultural mindset, personal status, level of education, and so on).

This study reports the results of the “Oncology in Motion” (OiM) initiative, developed within the National Cancer Institute of Aviano, Italy [24]. The OiM program was launched in 2018 as a co-production and telemedicine path dedicated to the rehabilitation and oncology education of women treated at the National Cancer Institute with a diagnosis of breast cancer. To our knowledge, OiM was the first oncology and post-surgical program entirely co-produced and co-designed, employing a multi-stakeholder approach and the use of multiple knowledge translation tools to foster cooperation [21,25,26]. Clinical professionals, patients, former patients, no–profit associations, scholars, and policymakers were involved in the project, sharing their ideas and contributions [25,26]. In the OiMprogram, following surgery, each eligible patient was offered a personalized rehabilitation and physical activity path, thanks to the OiM mobile app and wearable provided by the Institute. The project granted each patient a constant connection with specialized staff, who monitored, trained, and stimulated the correct execution of physical exercises at a distance. The OiM app collected data from Google Fit through the smartphone and wearable and transmitted these to the platform. Medical staff could access such information, set personalized step and cardio goals, constantly monitor progress, and send customized push-up notifications to users when necessary [27].

The primary objective of the study was to verify whether breast cancer patients, following surgery, perform physical activity if asked and educated to do so. Moreover, the study aimed to investigate the acceptance of the use of wearables to understand whether these tools could represent a valid path of education and encouragement for physical activity. The study reported the results of the OiM program, conceived within the National Cancer Institute of Aviano, Italy, and involved 144 breast-operated patients in a follow-up and rehabilitation phase after oncological surgery.

## 2. Methods

### 2.1. Type of the study and study population

The study is of an interventional pilot type, with a prospective, single-center cohort design, conducted at the National Cancer Institute - IRCCS CRO of Aviano, Italy [18–20]. The study received Ethical committee approval: CRO-2019-11 National Cancer Institute – IRCCS CRO Aviano, Italy.

The OiM study involved patients diagnosed with breast cancer undergoing elective breast surgery at the Institute in the period between March 2020 and March 2021. Breast surgery included quadrantectomy with the removal of the sentinel lymph node, mastectomy with and without axillary lymphadenectomy, and with and without contextual reconstructive surgery.

### 2.2. Inclusion/exclusion criteria

Patients had to meet the following study eligibility criteria:

- To have an age over 18.
- To feature an ASA status class I–III.
- To have the ability to express their informed consent to participate.
- To be available to conduct the scheduled periodic follow-up visits at the Institute.

Exclusion criteria included:

- Patients with cardiac pacemakers.
- Patients unable to perform post-surgical physical activity.
- Patients with heart disease and lung disease that contraindicate physical activity.
- Patients with insufficient digital literacy (ability to use a smartphone).
- Patients who refused to participate in the study (absence of signed informed consent).

### 2.3. Sample size

A total of 144 patients were enrolled in the program, excluding cases of withdrawal or declared complications. The Institute usually handles around 400 breast cancer patients per year. All of 144 participants were monitored for 12 months. In this first phase, data were collected during periodic visits scheduled 1, 3, 6, and 12 months after surgery. All enrolled patients signed informed consent for participation and data processing. After enrollment, each patient was given a watch (Kalenji on move 500 model) or a wearable band (Polar Fascia Cardio H9 model - Bluetooth and ANT+) and assigned a unique and anonymous alphanumeric ID. The data collection used anonymized data. Phone interviews were conducted after the end of the program to further deepen the results in terms of adherence and issues in training and using the technology.

### 2.4. Study protocol

The physical activity protocol included 150 min per week of aerobic activity (running or cycling, at the patient’s choice), with moderate intensity, that is, at a level of 13–14 (130–140 beats per minute) on the Borg scale, measured and recorded by the provided APP and sent to the operator.

All patients received specific training at the enrollment phase and after the download of the specific APP on their smartphone. All of them were supported to try several times all the functionality of the software tool [27].

Concerning physical activity, the enrolled patients were instructed by a kinesiologist trained in oncology physical activity. Follow-up visits was performed at 1,3,6,12 months after breast surgery. The patients were assessed during follow-up visits also with the aim of identifying any objective conditions that might have arisen after the intervention, potentially leading to non-adherence to the proposed physical activity.

The data sent were related to the minutes of daily aerobic activity, relative average heart rate during that part of the training (detected automatically), and number of strength and resistance training sessions (entered manually by the patients). Before each periodic meeting, the kinesiologist had access to this information to plan the meeting itself. During the follow-up visits, the kinesiologist modified the patients’ training schedule if some exercises were too difficult to perform, creating a personalized path that was effective and enjoyable for the patients. The kinesiologist was also available at any time between visits if necessary. Each periodic meeting was also an opportunity to check the study’s digital assessment.

### 2.5. Statistical analysis

The sample size was not calculated as this was a pilot study. Data were further analyzed through R (R-4.3.1 for Windows) [22]. A Whitney test was employed to check eventual differences in the two subsets: about those women who carried on physical activity and those who did not. Further analysis of the sample based on a Pearson’s chi-squared test of independence was carried out to analyze correlation between the type of surgical intervention and the physical activity performed.

Flow chart of the study is reported below in Table 1.

## 3. Results

The main results of the study are reported in the following Table 2.

### 3.1. Population characteristics and cause of poor physical activity adherence

**Fig. 1:**
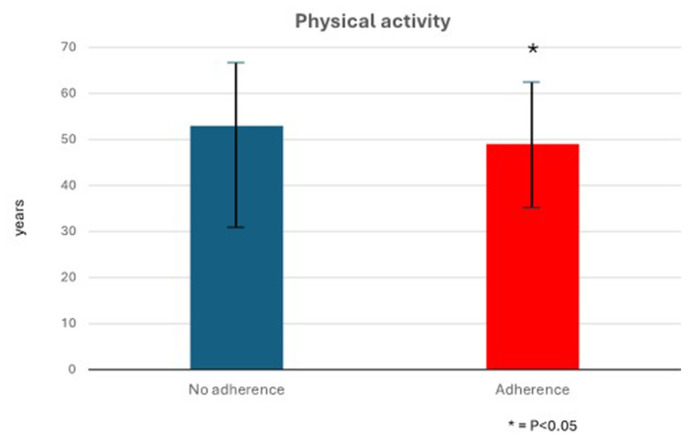
Study results – Age of patients versus physical activity performed. Correlation between age (expressed in years) and adherence or not adherence to proposed physical activity plan. * = p < 0.05 (statistical significance). Values are expressed as mean ± standard deviation.

As reported above, the enrolled patients were assessed during follow-up visits also with the aim of identifying any objective conditions that might have arisen after the intervention, potentially leading to non-adherence to the proposed physical activity. Still, none of the enrolled patients showed such problems. Concerning age, patients ranged from 31 to 69, with a mean age of 52. 76 of them underwent quadrantectomy, 62 had a mastectomy (Fig. 1).

Despite enrollment in the program and the subsequent onboarding [19], only 79 of them (equal to 55% of the sample) declared to be carrying on physical activity during the scheduled follow-up visits. Of these, 52 (equal to the 66% of women training regularly) were able to use the device, send data to the Institute, and receive push-up notifications and counseling. One-third of the women undergoing fitness activity were not able to record and send their progress.

Finally, all the women declaring that they were not training and those performing physical activity but not being able to send data to the Institute were asked to further explain the issues encountered in sticking to the program and personalized training path and in the use of wearables to record and submit their performance data and receive feedback. 84 participants agreed to provide the investigators with further details.

Most of the recorded difficulties were related to the use of technology (33 participants, equal to 39% of responses). 19 participants admitted that they were lazy and decided to stop the planned physical activity. 13 patients declared that they could not carry on or had to stop the personalized fitness program because of a worsening of their health conditions. 11 participants underlined that their family duties made adherence impossible, while 4 complained about work-related issues. Finally, 4 patients stated that they stopped because of a change in the referral institute, as Aviano was far away from their residence. 8 patients did not provide any reason. Data were further analyzed through R (R-4.3.1 for Windows) [22]. A Whitney test was employed to check eventual differences in the two subsets: about those women who carried on physical activity and those who did not. Results show that there is a statistically significant difference in the two subsets, where older people tend to perform less physical activity. The chart reported in Table 2 shows that patients who declared they did not follow the prescribed sports plan (53 *±* 16 years Versus 49 *±* 12 years) have a higher median age. Women sticking to the suggested training plan (group 1) have a lower median age. The age of the patients was normally distributed, and the p-value from the Student’s t-test for unpaired samples was 0.03 (significant). Tumor characteristics and postoperative factors were not analyzed in this context because, at each follow-up visit, the operator could identify any other potential causes of non-adherence to therapy.

**Fig. 2:**
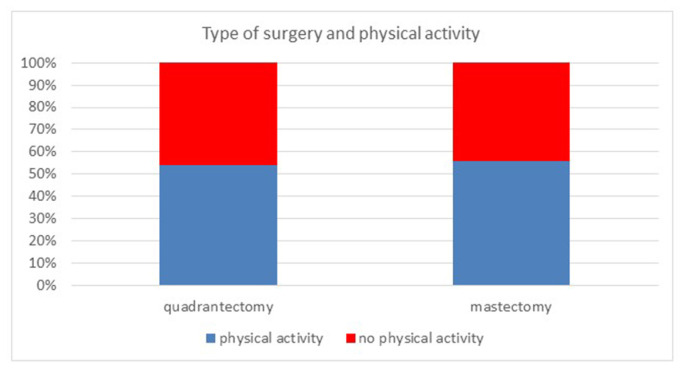
Correlation between physical activity and type of surgery. No statistically differences observed between “quadrantectomy group” and “mastectomy group” (p > 0.05).

Further analysis of the sample based on a Pearson’s chi-squared test of independence underlined how there is no significant correlation between the type of surgical intervention and the physical activity performed, recording a p-value of 0.2663 (*>*0.05) (Fig. 2).

A further analysis of the causes of no physical activity recording are resumed in Table 3. Data were analyzed with Chi-square test for each category.

The p-values at Chi – square test highlight the categories with statistically significant differences compared to the uniform distribution.

The data was analyzed by comparing each category (reason for non-physical activity) with all the others.

In particular:

- “Working duties” and “Change in the institute of reference” are highly significant (p < 0.0001).
- “Difficulties in the use of technology”, “Family duties”, “Worsening of health conditions”, and “No response” show significant differences with p < 0.05.
- “Laziness” does not show a significant difference (p *>* 0.05).

## 4. Discussion

### 4.1. Age and physical activity

Findings underline that the majority of patients who agreed to join the study are part of the age group ranging from 40 to 54 years. These results are in line with the statistics concerning the pathology [28]. People of this age group are expected to be young enough to perform physical activity in a proper and profitable way and to have enough digital literacy to use tools like smartphones and smartwatches. Indeed, data show a correlation between age and fitness activity, with older patients less willing to stick to the tailored sports plan. These results may not be surprising. Still, it should be highlighted how, according to the clinical protocol and study design [29], all eligible patients had to be fit enough to participate in the OiM program. Moreover, the OiM staff (professionals with a Master’s degree in sports sciences and experience in dealing with people with health issues or in a recovery phase) tailored the fitness schedule according to the specific patient, her personal situation, and the severity of the disease and the eventual side treatments (like chemo, radiation therapy, or pharmacological plans). Indeed, no statistically significant correlation was found between the type of surgery and the physical activity performed.

It should be noted that the smartwatch was provided for free by the Institute as a part of the program. Therefore, no costs or acquisition or access barriers were set on patients. Both situations (not respecting the fitness schedule or not using the device and the OiM app while training) were not ideal. Indeed, women not performing physical activity would miss the opportunity to improve their healing and post-surgical recovery, as the international literature agrees on the crucial role of sports for oncological patients [3,4], especially when these programs include a tailored approach [12,13]. Women not using devices would not enjoy a privileged connection with the medical staff of the Institute, and receive personalized notifications and feedback on the results recorded in terms of compliance, outcomes, risks, and benefits [27]. Moreover, these patients would not contribute to creating collective knowledge about the rehabilitation paths by providing their data to be analyzed in an aggregated form.

### 4.2. Motivations of the patients

Reflections should be focused on the motivations of the patients not performing physical activity or not using the provided wearable and app. Results show a problematic relationship with technology, which is worth deepening with further interviews. Interestingly, the mean age of patients declaring such issues was 54, ranging from 34 to 69. It emerges a worrying lack of technological education and acceptance, especially when considering the age group of the patients. This relevant issue should be taken care of by the Institute during the onboarding phase and follow-up visits. Clinical education should, therefore, be coupled with technological support and counseling, as reported and recommended by previous investigations [23,24].

Another relevant recorded cause of physical inactivity is laziness. This issue may be resolved or, at least, mitigated with dedicated education plans to stimulate the understanding of the importance of sports in cancer recovery and the opportunity to enjoy a tailored plan with the help of technology. Adequate knowledge translation facilitators [20,20] are recommended to engage the patients and let them understand sports’ powerful and positive role in speeding the healing and recovery process. Such facilitators may include the experience of testimonials and former patients [30], magazine and blog publications, brochures, leaflets, and billboards at the Institute [21,25]. However, even the role of nursing professionals or case managers may look central, as they spend more time with the patients, and they usually enjoy their complete trust [26,31].

Other causes reported by patients as limitations in sticking to the defined fitness activities and in the use of wearables are health complications, family issues, and work duties. All these causes may look more challenging to solve using a proper educational approach, especially in the case of a worsening of the health condition. Still, it should be noted the full availability of fitness professionals like physiotherapists and graduated fitness experts to reshape the customized fitness plan [12,13]according to the new situation and the emerging patient’s needs.

Finally, a change in the recovery strategy due to a different referral center (mainly because of the need to be followed up by a spoke center closer to home) may compromise the encouraging results obtained by sticking to the physical activity program. More collaboration and knowledge sharing between cancer institutes and hospitals (even those smaller ones that serve as spokes to encourage patients’ proximity) should be encouraged to promote sports education and the use of technological instruments.

## 5. Conclusions

Although the literature underlines the importance of personalized training paths in cancer recovery, results from the OiM project [25–27] record low adherence and several issues in sticking to the plan and using technology. Patient education and counseling should, therefore, be enhanced to stimulate physical activity and overcome eventual barriers to the use of technological instruments. This is a challenging strategy that requires a multidisciplinary effort and the use of multiple knowledge translation tools and facilitators to engage patients and make them aware of the potential positive role of the fitness and technology combo.

As with all the studies, ours has limitations. First of all, the study design not differentiate between those female patients who already practiced regular physical activity before surgery and at what level, and patients who started from scratch with the provided exercise program. Therefore, we were unable to conduct specific sub-analyses, which could have been particularly valuable. Furthermore, we did not enroll a group of women who were not given devices. All patients were instructed about the importance of physical activity as a prevention and healing opportunity. All of them were provided with a personalized path. Still, the follow-up protocol and interview structure was the same for all the eligible participants. Therefore, the information obtained refers to the sum of the effects of digitalization and the coaching program provided by the Institute.

The information provided by the patients during the investigation needs further explanation to dig deeper into the causes that prevent women from sticking to the tailored fitness plan and using the device to connect with the referral center. As no technology acquisition barriers exist (as the smartwatch or bend was provided for free as part of the program), it would be crucial to understand, for instance, what kind of issues people experience with technology: lack of general digital literacy, matters connected with the specific use of the tool, lack in trust, privacy concerns, … Other types of difficulties are also worth deepening, for example, those associated with family-related duties, such as lack of time, lack of support, … Only a detailed understanding of the barriers and problems can foster the study of tailored solutions and strategies to support these patients in getting the best out of tools (like sports and e-health) to facilitate their healing journey. An in-depth qualitative approach through one-to-one semi-structured interviews is recommended for a more comprehensive understanding of the phenomenon.

Moreover, further sample analysis may lead to new research outcomes concerning the (better) prognosis or psychological health of those who engaged in physical activity or used the devices.

Low technological education was a primary obstacle to proper data collection. This may be due to the fact that, while data on aerobic activity were automatically extracted from the app, data on strength and resistance activity had to be manually entered by the patient from her smartphone. For future studies, using on/off devices with entirely automatic data transmission might help overcome the related barriers.

## Figures and Tables

**Table 1:** Study flow chart.

Study flow chart
Type of study: prospective, pilot, interventional Surgery of choice: quadrantectomy with sentinel lymph node, mastectomy with and without axillary lymphadenectomy, with and without contextual reconstructive surgery Inclusion criteria: Age over 18, ASA status class I–III, Ability to express their informed consent to participate, Available to conduct the scheduled periodic follow-up visits at the Institute. Eclusion criteria: cardiac pacemakers, unable to perform post-surgical physical activity, heart disease and lung disease that contraindicate physical activity, insufficient digital literacy refused to participate in the study Follow upvisits: 1,3,6, 12 months after breast surgery

**Table 2:** Study results – General findings.

Variable	Measure	
Totale number of enrolled patients	144	100%
Age at the time of enrollment		
Mean	51	
Standard deviation	9	
Youngest age	31	
Oldest age	69	
Type of surgical intervention		
Quadrantectomy	78	54,16%
Mastectomy	66	45,84%
Physical activity declared at the time of follow-ups		
Yes	79	54,86%
data successfully submitted	52	65,82%
data not submitted	27	34,18%
No	65	45,14%
Difficulties recorded	84	58,33%
Difficulties in the use of technology	33	39,29%
Laziness	19	22,62%
Worsening of health conditions	13	15,48%
Family duties	11	13,10%
Working duties	4	4,76%
Change in the institute of reference	4	4,76%
No response	8	–

**Table 3:** Causes of no physical activity recognition.

Category	Chi^2^	p value	* = statistically significance
Difficulties in the use of technology	6.29	0.0122	yes
Laziness	0.47	0.4941	no
Worsening of health conditions	4.21	0.0402	yes
Family duties	6.29	0.0122	yes
Working duties	16.83	4.09 × 10^−5^	yes
Change in the institute of reference	16.83	4.09 × 10^−5^	yes
No response	10.18	0.0014	yes
